# Dr. Boisseau

**Published:** 1837-01

**Authors:** 


### OBITUARY.

### DR. BOISSEAU.

Lately at Metz, aged 43, François Gabriel Boisseau, M.D., a distinguished writer, and the author of several works of great merit. Dr. Boisseau was born at Brest, on the 12th of October, 1791. He entered at an early age into the army, and served in Spain and in Germany from 1810 to 1813. At the time of the first abdication of Napoleon, he was prisoner at Dresden, and returned to France with the liberated garrison. He joined the army on the return of Napoleon from Elba, and after the battle of Waterloo was appointed Assistant to the military establishment at Val de Grâce. M. Boisseau returned to his medical studies with great ardour, and with proportionate success. In 1817, he obtained the public prizes given at Val de Grâce, and in the spring of the same year took the degree of Doctor of Medicine. During his residence at the Val de Grâce, Boisseau was the most distinguished and most philosophical advocate of the doctrines of Broussais, which he advanced and defended by his speeches and numerous writings, with singular force and effect. After the period of his graduation, he pursued his literary labours with undiminished zeal, and published many works of great merit. After the revolution in July 1830, he was appointed Physician or Professor of the Military Hospital for Education at Metz, where he died on the 2d of January, 1836.

The following is a list of the principal writings of Dr. Boisseau: *Pyrétologie physiologique, ou Traité des Fièvres*, 1 vol. 8vo.; *Nosographie Organique, ou Traitè de Médecine pratique*, 4 vols. 8vo.; all the medical articles of the *Dictionnaire abrégé des Sciences Médicales*, 15 vols. 8vo.; numerous articles in the *Biographie Médicale*, the *Encyclopédie Moderne*, the *Journal Hebdomodaire*, &c. He edited and annotated the following works: The works of Pujol; Dr. Thomson's Treatise on Inflammation; Rolando's Physiological Inductions; Tissot's Treatise on the Health of Literary Persons. He was for twelve years principal editor of the *Journal Universel des Sciences Médicales*.

Like so many members of our profession, who devote themselves to the literary and scientific part of medicine, Dr. Boisseau too much neglected the advancement of his private fortune. He has consequently died very poor, leaving a widow and three children unprovided for, and for whom a subscription is now opened among the members of the profession in Paris.

---

